# The Danish Out‐of‐Hospital Cardiac Arrest Trial: A Statistical Analysis Plan

**DOI:** 10.1111/aas.70288

**Published:** 2026-06-23

**Authors:** Simon Mølstrøm, Jacob E. Møller, Henrik Schmidt, Anders M. Grejs, Steffen Christensen, Jesper Kjærgaard, Christian Hassager, Martin A. S. Meyer, Lars P. K. Andersen, Bodil S. Rasmussen, Jo B. Andreasen, Sören Möller

**Affiliations:** ^1^ Department of Anesthesiology and Intensive Care Odense University Hospital Odense Denmark; ^2^ Department of Clinical Research, Faculty of Health Sciences University of Southern Denmark Odense Denmark; ^3^ Department of Cardiology Odense University Hospital Odense Denmark; ^4^ Department of Cardiology, the Heart Centre Copenhagen University Hospital—Rigshospitalet Copenhagen Denmark; ^5^ Department of Intensive Care Aarhus University Hospital Aarhus Denmark; ^6^ Department of Clinical Medicine, Faculty of Health Aarhus University Aarhus Denmark; ^7^ Department of Clinical Medicine, Faculty of Health Sciences University of Copenhagen Copenhagen Denmark; ^8^ Department of Intensive Care Zealand University Hospital, Koege Hospital Køge Denmark; ^9^ Department of Anesthesiology and Intensive Care Aalborg University Hospital Aalborg Denmark; ^10^ Department of Clinical Medicine, Faculty of Medicine Aalborg University Aalborg Denmark; ^11^ Epidemiology, Biostatistics and Biodemography, Department of Public Health University of Southern Denmark Odense Denmark

**Keywords:** backrest elevation, DANOHCA trial, dexamethasone, olanzapine, post‐cardiac arrest care, sedation, statistical analysis

## Abstract

**Background:**

Post‐cardiac arrest care for patients resuscitated from out‐of‐hospital cardiac arrest (OHCA) includes multiple pharmacological and physiological interventions, yet optimal strategies to reduce post‐cardiac arrest syndrome–related morbidity and mortality remain uncertain. The DANOHCA (Danish Out‐of‐Hospital Cardiac Arrest) trial evaluates four such interventions—high‐dose dexamethasone, prophylactic olanzapine, elevated backrest position, and early wakeup—in a factorial trial framework. This manuscript presents the complete statistical analysis plan for analyses common to all four DANOHCA interventions.

**Methods:**

DANOHCA is an investigator‐initiated, multicenter, randomized, 2 × 2 × 2 × 2 factorial clinical trial enrolling comatose adult OHCA patients with a presumed cardiac etiology who achieve sustained return of spontaneous circulation, with randomization within 180 min of return of spontaneous circulation. Participants are co‐randomized to Dexamethasone versus placebo, olanzapine versus placebo, backrest elevation at 35° versus 5°, and early (≤ 6 h) versus late (28–36 h) wakeup, with pharmacological interventions blinded and physiological interventions open‐label. This statistical analysis plan specifies the primary and secondary outcomes, covariate adjustment, handling of missing data, assessments of interactions between interventions, and assumption checks, with all primary analyses conducted according to the intention‐to‐treat principle. The analysis plan is finalized prior to completion of randomization.

**Results:**

The primary outcomes are 90‐day all‐cause mortality for the dexamethasone and backrest interventions, and days alive outside hospital within 30 days for the olanzapine and early wakeup interventions, with common secondary and tertiary endpoints including mortality at later time points, neurological function, health‐related quality of life, organ function, and safety outcomes. Mixed‐effects regression models, Cox regression, stratified nonparametric tests, and prespecified sensitivity analyses will be applied to minimize bias and quantify intervention effects, including their associated uncertainties.

**Conclusion:**

This predefined statistical analysis plan provides a detailed, transparent framework for the primary analyses and reporting of the DANOHCA trial.

**Trial Registration:** Clinical Trials Information System (CTIS): 2024‐515997‐28‐00

## 1 Background

Critical care management after out‐of‐hospital cardiac arrest (OHCA) aims to limit ischemia–reperfusion injury by targeting sedation, hemodynamics, ventilation, and temperature, but optimal strategies to reduce post‐cardiac arrest syndrome‐related morbidity and mortality remain uncertain [1, 2]. Post‐cardiac arrest syndrome comprises a systemic inflammatory response, cerebral and myocardial dysfunction, and the underlying precipitating pathology [3]. It results in high mortality rates and long‐term neurological disability despite contemporary intensive care, including temperature control and organ support. Several potentially modifiable treatment domains in the early post‐resuscitation phase—systemic inflammation, cerebral perfusion and intracranial pressure, duration of sedation, and delirium—are supported by expert consensus based on pathophysiological rationales and observational data but have not been evaluated in large, pragmatic, randomized trials in comatose patients resuscitated from OHCA.

The DANOHCA (Danish Out‐of‐Hospital Cardiac Arrest) trial is an investigator‐initiated, randomized, controlled, multicenter, 2 × 2 × 2 × 2 factorial clinical trial evaluating four distinct interventions in patients who remain comatose after resuscitation from OHCA.

The trial evaluates two pharmacological interventions, that is, high‐dose dexamethasone to control systemic inflammation and prophylactic olanzapine to prevent delirium, and two physiological interventions, that is, elevated backrest positioning to improve cerebral perfusion pressure and early versus late wakeup call to reduce exposure to deep sedation and facilitate earlier hospital discharge. In general intensive care, current guidelines advocate minimal sedation and daily wakeup calls [4]. However, in post‐cardiac arrest care, prolonged deep sedation for 24–48 h has been standard of care since the introduction of targeted temperature management, despite limited evidence [2, 5, 6]. The early wakeup intervention tests whether the principle of minimal sedation also applies after cardiac arrest.

These four interventions target distinct but complementary mechanisms in early post‐resuscitation care, systemic inflammation, delirium, cerebral perfusion, and sedation strategy, aiming collectively to reduce morbidity and mortality after cardiac arrest.

All patients are randomized in a 1:1 factorial design to all four interventions simultaneously.

The four hypotheses are:

*There is no difference in 90‐day all‐cause mortality between the dexamethasone and placebo groups*.

*Treatment with dexamethasone leads to improved rates of survival at 90 days compared to placebo*.

*There is no difference in 90‐day all‐cause mortality between backrest elevation at 35° and 5°*.

*An elevated backrest at 35° leads to improved rates of survival at 90 days compared to elevation at 5°*.

*There is no difference in days alive outside of hospital within 30 days between early wakeup (sedation ≤ 6 h) and mandatory sedation (28–36 h)*.

*Mandatory sedation for 28–36 h leads to more days alive outside of the hospital in 30 days compared to sedation for ≤ 6 h*.

*There is no difference in days alive outside of hospital within 30 days between Olanzapine and placebo*.

*Treatment with olanzapine leads to more days alive outside of the hospital in 30 days compared to placebo*.

This statistical analysis plan is one of several companion manuscripts for the DANOHCA trial, each addressing a prespecified intervention within the shared factorial trial framework. Intervention‐specific rationale and procedures are described in four corresponding protocol manuscripts; the present SAP outlines the methodological approach to analyzing the common primary, secondary, and tertiary outcomes of the DANOHCA trial. This predefined plan aims to minimize analytic bias, ensure transparency, and enhance the interpretability of intervention effects on clinically important outcomes.

## 2 Methods

The DANOHCA trial is registered with the Clinical Trials Information System (CTIS) under trial number 2024‐515997‐28‐00. The design and interventions of the DANOHCA trial are described in detail in four dedicated protocol papers. Patients and members of the public were not involved in trial design, given the emergency circumstances of out‐of‐hospital cardiac arrest and patients' inability to participate in planning. The primary and secondary outcomes were selected to reflect priorities relevant to patients and their families, including survival and time alive outside the hospital.

In summary, the trial will enroll adults (≥ 18 years) admitted after OHCA with return of spontaneous circulation (ROSC). Patients will be eligible if they fulfill all predefined inclusion criteria and none of the exclusion criteria.

### 2.1 Inclusion Criteria

- Out‐of‐hospital cardiac arrest of presumed cardiac cause.
- Age ≥ 18 years.
- Sustained ROSC (defined as ≥ 20 min without chest compressions or mechanical circulatory support).
- Unconsciousness (Glasgow Coma Scale < 9, unable to obey verbal commands) upon admission.

### 2.2 Exclusion Criteria

- Presumed noncardiac cause of arrest.
- Unwitnessed asystole.
- Suspected or confirmed intracranial bleeding or stroke.
- Pregnancy.
- Admission temperature < 30°C.
- Persistent cardiogenic shock (systolic blood pressure < 80 mmHg despite fluid loading and vasopressor or inotropic medication).
- Known disease making 180‐day survival unlikely.
- Known pre‐arrest Cerebral Performance Category 3 or 4.
- > 180 min from ROSC to randomization.

### 2.3 Randomization and Blinding

Randomization is performed in a 1:1 factorial design for all four interventions, using sealed intervention boxes containing study drugs and written instructions. Randomization is generated with varying block sizes to ensure balance across the 16 possible intervention combinations. The balance of allocation across the 16 possible combinations is monitored when generating the randomization sequence and as part of the planned interim analyses.

Pharmacologic interventions (dexamethasone and olanzapine) are double‐blinded, with study drugs prepared by the pharmacy in the Capital Region of Denmark and administered by nurses uninvolved in the patient's care. Physiologic interventions (backrest elevation and early wakeup) are open‐label because blinding them is inherently difficult. Outcome assessors, statisticians, data managers, the steering group, and the data safety and monitoring committee will be blinded to treatment allocation.

### 2.4 Interventions

#### 2.4.1 The Dexamethasone Intervention

Patients randomized to Dexamethasone receive 20 mg intravenously (administered as Dexamethasone phosphate) over 15 min as soon as possible after admission, followed by 20 mg intravenously daily at 0600 h (or at least 8 h after the initial dose) for two additional days, for a total of three doses. Patients in the control group receive a matching placebo (sodium chloride).

#### 2.4.2 The Olanzapine Intervention

Patients randomized to olanzapine receive 10 mg via nasogastric tube as soon as possible after admission, followed by 10 mg daily at 6 PM (with a minimum of 12 h between doses) for two additional evenings, for a total of three doses or until intensive care unit discharge, whichever occurs first. Patients in the control group receive a matching placebo. All patients are monitored with mandatory telemetry for 96 h or until withdrawal of life‐sustaining therapies to assess for QT prolongation and arrhythmias.

#### 2.4.3 The Backrest Elevation Intervention

Patients are randomized to either backrest elevation to 35° in the semi‐Fowler position (with the lower limb elevated) or backrest elevation to 5° during the initial 72 h or until extubation, whichever occurs first. Adherence to the assigned position is assessed every 8 h. The intervention could be temporarily discontinued for procedures or mobilization, but resume if invasive ventilation continues.

#### 2.4.4 Early Wakeup Intervention

Patients are randomized to an early wakeup call after ≤ 6 h or a late wakeup call between 28 and 36 h after intensive care unit admission. Criteria for extubation readiness include Glasgow Coma Scale ≥ 12, Richmond Agitation‐Sedation Scale 0 to −1, ability to raise arm or voluntary handshake on command, successful spontaneous breathing trial, and low ventilator settings (pressure support ≤ 14 cm H_2_0 (or lower according to local practice), positive end‐expiratory pressure ≤ 8 cm H_2_O [10 cm H_2_O if obese], and fraction of inspired oxygen ≤ 40%). A wakeup call can be aborted in case of seizures, respiratory distress, shock, or other clinical reasons. Sedatives before the scheduled wake‐up call are permitted as needed for clinical care in the early‐wake‐up call group and are mandatory in the late wake‐up call group.

### 2.5 Outcomes

#### 2.5.1 Primary Outcomes

The trial employs intervention‐specific primary outcomes:

- Dexamethasone intervention: All‐cause mortality at 90 days.
- Backrest elevation intervention: All‐cause mortality at 90 days.
- Olanzapine intervention: Days alive outside hospital within 30 days.
- Early wakeup intervention: Days alive outside the hospital within 30 days.

The choice of intervention‐specific primary outcomes reflects the anticipated mechanism of action for each intervention. Dexamethasone and backrest elevation target pathophysiological pathways that may directly affect early survival—dampening systemic inflammation and increasing cerebral perfusion pressure, respectively—thereby making 90‐day all‐cause mortality the most clinically relevant primary endpoint. In contrast, olanzapine and the early wakeup strategy primarily aim to reduce delirium and sedation‐related complications, translating into improved functional recovery and shorter hospital stays rather than a direct effect on mortality. Days alive outside the hospital within 30 days reflect both survival and time to recovery, and are therefore a more sensitive and clinically meaningful primary endpoint for these two interventions.

Days alive outside the hospital within 30 days are calculated by subtracting the time spent in the hospital or deceased within 30 days, then rounding down to the nearest whole number. Thus, a patient dying on admission day has zero days alive outside the hospital, and a patient with hospitalization exceeding 30 days also has zero days alive outside the hospital. A day (≥ 24 h) spent at a hospital or healthcare facility operated by a Danish region counts as time in hospital, whereas outpatient visits and brief hospitalizations < 24 h count as days alive outside hospital.

#### 2.5.2 Secondary Outcomes

The following secondary outcomes are assessed across all four interventions. Intervention‐specific secondary outcomes are detailed in the four dedicated protocol publications.

Common secondary outcomes for all four interventions include:

- All‐cause mortality at 90 days (for interventions where this is not the primary outcome).
- Days alive outside hospital within 30 days (for interventions where this is not the primary outcome).
- Cerebral Performance Category and modified Rankin Scale at follow‐up 3–6 months after cardiac arrest.

#### 2.5.3 Exploratory (Tertiary) Outcomes

- All‐cause mortality at 180 days and 1 year.
- Inflammatory and organ markers during the initial 72 h.
- Neurofilament light chain and neuron‐specific enolase at 0, 24, 48, and 72 h.
- Cognitive function assessment (Repeatable Battery for the Assessment of Neuropsychological Status, Trail Making Test, and Montreal Cognitive Assessment) at 3–6 months.

### 2.6 Sample Size

The trial aims to include 1000 patients across five sites, covering the entire Danish population. Sample size calculations were performed separately for each intervention based on its primary outcome.

For the Dexamethasone and backrest elevation interventions (primary outcome: 90‐day mortality), assuming an overall mortality of 35% and a 25% relative risk reduction (to 26% mortality), with 85% power and a two‐sided significance level of 5%, 468 patients per group are required. To account for potential dropouts and loss to follow‐up, 500 patients per group (1000 total) are planned.

For the olanzapine and early wakeup interventions (primary outcome: days alive outside hospital within 30 days), estimates assumed 35% mortality during initial hospitalization (0 days alive outside hospital) and a mean of 15 days outside hospital for survivors, yielding a population mean of 9.8 days with an estimated standard deviation of 7 days. To detect an increase of 1.5 hospital‐free days with > 90% power and a two‐sided significance level of 5%, a total of 920 patients were required.

The combined sample size of 1000 patients provides adequate power for all four primary outcomes within the factorial design. The trial is not powered to detect interactions between interventions; prespecified interaction analyses (Section 3.5) are exploratory and hypothesis‐generating.

## 3 Statistical Analysis Principles

### 3.1 General Principles

All analyses will be conducted according to the modified intention‐to‐treat principle, including all randomized participants who meet the inclusion criteria and have available outcome data, or imputed outcome data (see missing data section below). Participants who do not fulfill the inclusion criteria will be excluded from analyses. If consent is subsequently withdrawn, data collected prior to withdrawal will be included in the primary analysis in accordance with the EU Clinical Trials Regulations, unless the participant explicitly requests deletion of their data. A per‐protocol analysis will be performed as a sensitivity analysis, including only participants who received all scheduled doses of pharmacologic interventions, adhered to the assigned backrest position for ≥ 80% of the time, and had a wakeup call attempted within the assigned time frames.

Data will be analyzed and reported as four individual trials, with no adjustment for multiple comparisons planned. This approach is justified because almost all previous trials in the hospital phase of out‐of‐hospital cardiac arrest care have been neutral, and all four interventions are accepted without considering potential interactions. If more than one intervention shows a non‐neutral result, interactions between interventions will be evaluated by including pairwise and higher‐order interaction terms in the primary regression models for each intervention, applying the prespecified thresholds defined in Section 3.5 (interaction *p* < 0.01 and absolute effect ≥ 5.6%). If a clinically important interaction is identified, results for the affected interventions will be presented both as overall estimates and stratified by the interacting intervention allocation. All regression analyses will be adjusted for site as a covariate.

All statistical significance tests will be two‐sided, with a significance level of 5%. No alpha spending or adjustment of the final significance level is required, as the planned interim analysis at 500 enrolled patients is a safety review conducted by the independent data‐monitoring committee without formal efficacy or futility testing (see Section 3.9). Throughout, categorical variables will be presented as counts with proportions, whereas continuous variables will be presented as means ± standard deviations for normally distributed variables or medians (25th–75th percentiles) for nonnormally distributed variables. The normal distribution will be evaluated using quantile‐quantile plots.

### 3.2 Analysis of Primary Outcomes

#### 3.2.1 Mortality Outcomes (Dexamethasone and Backrest Interventions)

The primary analysis for the Dexamethasone and elevated backrest interventions will use log‐binomial regression to estimate the relative risk of 90‐day mortality, defined as the proportion of patients who die from any cause between randomization and day 90. This will also be reported for the early wake‐up and olanzapine interventions. If log‐binomial models are infeasible due to non‐convergence, logistic regression models will be used instead, and OR will be reported instead of RR. As additional analyses, Kaplan–Meier curves for each allocation group will be estimated, graphically displayed, and compared by the log‐rank test. Furthermore, Cox proportional hazards models will be applied to estimate hazard ratios with 95% confidence intervals for time to death between treatment groups as a secondary outcome for all four interventions. As prespecified subgroup analyses, the interaction between treatment allocation and each of the following baseline variables will be assessed: sex, age, time to ROSC, lactate level upon hospital admission, shockable primary rhythm, witnessed cardiac arrest, bystander‐performed CPR, and presence of STEMI consistent with acute myocardial infarction. Proportional hazards assumptions will be assessed using Schoenfeld residuals and log–log plots. If deviations from the proportional hazards assumption are detected, the relevant covariates will be included with time‐varying HR instead, or if this is not sufficient to solve the deviation, Cox regressions with stratified baseline hazards will be utilized. Further, causes of death (cardiovascular, neurological, multi‐organ failure) between the treatment arms will be compared as competing risks with the Nelson‐Aalen cumulative incidence method as hypothesis generating. Any changes from the pre‐specified analysis plan will be reported.

#### 3.2.2 Days Alive Outside Hospital (Olanzapine and Early Wakeup Interventions)

Days alive outside the hospital within 30 days will be analyzed using the Wilcoxon rank‐sum test between the two treatment arms. Results will be presented as medians with interquartile ranges and 95% confidence intervals for the median difference (calculated using non‐parametric bootstrapping with 1000 resamples). Relative differences between days alive outside hospital groups will be reported as percentages, with confidence intervals obtained by bootstrapping. As a prespecified supplementary analysis, days alive outside the hospital within 30 days will also be expressed as the mean between‐group difference with a 95% confidence interval. Sensitivity analyses using mixed‐effects linear regression models with site as a random effect, adjusting for other interventions, will be performed to assess the robustness of the findings.

### 3.3 Analysis of Secondary Outcomes

#### 3.3.1 Dichotomous Outcomes

No secondary or tertiary endpoints are adjudicated by a blinded endpoint committee. Dichotomous outcomes (mortality at other time points, unconsciousness at 96 h, and Confusion Assessment Method for the Intensive Care Unit positivity) will be analyzed using the chi‐square or Fisher's exact test, as appropriate. Results will be presented as proportions with risk ratios and 95% confidence intervals. Mixed‐effects logistic regression models with site as a random effect and other interventions as fixed effects will be used for adjusted analyses.

#### 3.3.2 Continuous Outcomes

Continuous endpoints assessed at multiple time points will be analyzed using linear mixed models, including a random intercept for each patient and a random effect for site. The treatment‐by‐time interaction will indicate whether the endpoint changes differently over time in the intervention versus control groups. Group differences will be reported as relative percentage differences. A logarithmic transformation will be applied to approximate a normal distribution as appropriate. Non‐parametric bootstrapping with 1000 resamples will be applied in case of deviations from distributional assumptions.

Continuous outcomes measured at a single time point will be analyzed using mixed‐effects linear regression with site as a random effect and other interventions as fixed effects. Results will be presented as mean differences with 95% confidence intervals.

#### 3.3.3 Count Data

Count data (intensive care unit length of stay, hospital length of stay, duration of intubation, days without delirium treatment) will be analyzed using the Wilcoxon rank‐sum test. Results will be presented as medians with interquartile ranges and 95% confidence intervals for the median difference obtained by bootstrapping.

#### 3.3.4 Ordinal Outcomes

Cerebral Performance Category and modified Rankin Scale at discharge and follow‐up will be analyzed as ordinal outcomes using proportional odds models with robust standard errors clustered by site and other interventions as fixed effects. Results will be reported as odds ratios with 95% confidence intervals. Proportional odds assumptions will be assessed using Brant tests.

### 3.4 Subgroup Analyses

Prespecified subgroup analyses will be performed for the following variables:

- Age (higher or lower than median).
- Sex (male vs. female).
- Initial rhythm (shockable vs. non‐shockable).
- Time to ROSC (higher or lower than median).
- Witnessed arrest (yes vs. no).
- Bystander cardiopulmonary resuscitation (yes vs. no).
- Presence of ST‐segment elevation myocardial infarction on admission (yes vs. no).
- Lactate level on admission (higher or lower than median).

Subgroup analyses will be performed using regression‐based tests for interaction. Results will be interpreted cautiously, given the risk of type I error with multiple comparisons. No formal adjustment for multiplicity will be applied, but findings will be clearly labeled as exploratory.

### 3.5 Assessment of Interactions Between Interventions

As a safety measure and to investigate potential synergistic or antagonistic effects, interactions among the four interventions will be assessed with respect to 90‐day mortality, days alive outside the hospital within 30 days, and the occurrence of serious adverse events. Interaction terms will be included in regression models, and interactions will be considered significant if the interaction term has *p* < 0.01 and demonstrates a clinically important effect (based on the anticipated intervention effect used in sample size calculations, i.e., absolute risk reduction of 5.6%).

### 3.6 Handling of Missing Data

Missing data for the primary outcomes are expected to be minimal (< 5%) given the registry‐based follow‐up for mortality and comprehensive hospital records for days alive outside the hospital. The primary analysis will include all participants with available data.

If missing data exceed 5% for primary outcomes and are believed to be missing at random, the following approaches will be used:

1. Multiple imputation: Missing values will be imputed using chained equations with 50 imputations, incorporating baseline characteristics and treatment allocation to the four interventions, but without including treatment interactions.
2. Best‐worst and worst‐best case scenarios: To assess the potential range of impact of missing data, best‐worst scenarios (all patients with missing data in the intervention group assumed a beneficial outcome; all in the control group assumed a harmful outcome) and worst‐best scenarios (reverse assumptions) will be presented. For numerical outcomes, best, respectively, worst‐case scenarios will be imputed using the minimum, respectively, maximum taken of the numerical outcome over all non‐missing patients.
3. Sensitivity analyses: Additional sensitivity analyses assuming all participants with missing data were either all alive or all dead (for mortality outcomes) will be performed.

For secondary and tertiary outcomes, missing data mechanisms will be evaluated, and multiple imputation will be used if appropriate. Complete case analyses will be reported alongside imputed analyses.

### 3.7 Assessment of Statistical Assumptions

All statistical analyses will undergo a systematic assessment of underlying assumptions [10][11].

#### 3.7.1 Assumptions for Regression Models

##### 3.7.1.1 Mixed‐Effects Models

For mixed‐effects logistic and linear regression models, the following assumptions will be assessed:

- Normality of residuals (linear models): Assessed using quantile‐quantile plots of residuals.
- Homogeneity of variances (linear models): Assessed by plotting residuals against covariates and fitted values.
- Random effects structure: Appropriateness of site as a random effect will be assessed using likelihood ratio tests. Normality of random effect estimates will be assessed using quantile‐quantile plots.

If assumptions are violated, appropriate transformations (logarithmic, square root), robust standard errors, or bootstrapping will be considered.

##### 3.7.1.2 Cox Proportional Hazards Models

Proportional hazards assumptions will be assessed using:

- Schoenfeld residuals plotted over time.
- Log–log plots of survival curves.
- Statistical tests of Schoenfeld residuals.

If proportional hazards assumptions are violated, time‐varying coefficients or stratified Cox models will be considered.

##### 3.7.1.3 Proportional Odds Models

The proportional odds assumption for ordinal outcomes will be assessed using:

- Brant tests for the parallel regression assumption.
- Graphical assessment of cumulative probabilities.

If assumptions are violated, partial proportional odds models or generalized ordered logit models will be used.

### 3.8 Safety Analyses

Adverse events and serious adverse events will be summarized by intervention group using descriptive statistics. The following adverse events will be specifically monitored: major bleeding, infections (pneumonia, sepsis, pyelonephritis, upper airway infections), renal impairment requiring renal replacement therapy, electrolyte disorders, metabolic disorders (hypoglycemia, prolonged hyperglycemia), cardiac events (ventricular fibrillation, ventricular tachycardia, atrial fibrillation requiring cardioversion, new need for pacing, prolonged QT interval > 560 ms, circulatory collapse), respiratory events (auto‐extubation, need for reintubation), seizures (tonic–clonic, myoclonic, status epilepticus), extrapyramidal side effects, skin complications (pressure ulcers, acne, atrophy, purpura, reduced wound healing), and death after withdrawal of life‐sustaining therapy.

Adverse reactions potentially related to specific interventions will be identified:

- Dexamethasone: Infections, hyperglycemia, skin complications (acne, atrophy, purpura, reduced wound healing).
- Olanzapine: Ventricular arrhythmias, prolonged QT interval, bucco‐linguo‐masticatory syndrome.
- Backrest elevation: Pressure ulcers occurring within 96 h.

Safety outcomes will be analyzed using descriptive statistics and risk ratios with 95% confidence intervals. No formal hypothesis testing will be performed for safety outcomes, but clinically meaningful differences will be highlighted.

### 3.9 Data Monitoring

An independent data‐monitoring committee comprising three clinicians with expertise in cardiac arrest and critical care research, and with no conflicts of interest, will oversee trial safety. The data‐monitoring and safety committee conducted a prespecified safety review after enrolment of 500 patients and recommended that the trial continue unchanged. No modifications to the protocol or sample size were made as a result of this review.

### 3.10 Quality Control and Adherence to GCP

The trial is monitored by the regional Good Clinical Practice monitoring unit, which will have full access to all data. A thorough monitoring plan was developed before the trial commenced.

### 3.11 Software

Statistical analyses will be performed using Stata version 18 or later (StataCorp, College Station, TX). All analyses will be fully reproducible with documented code. Analyses that include stochasticity (e.g., bootstrapping and multiple imputation) will be performed using a seed for pseudo‐random number generation.

### 3.12 Reporting

Results will be reported according to the Consolidated Standards of Reporting Trials (CONSORT) extension for factorial trials. Flow diagrams will document participant screening, randomization, follow‐up, and analysis for each intervention. Baseline characteristics will be presented by intervention allocation. Outcomes will be presented for each intervention, with effect estimates and 95% confidence intervals. Prespecified sensitivity and subgroup analyses will be clearly labeled.

Any deviations from this statistical analysis plan will be documented and justified in the final manuscripts. The steering committee will interpret the results, with treatment allocation coded until the analyses and conclusions are accepted. After acceptance, the trial will be unblinded. Results will be submitted for publication in peer‐reviewed journals regardless of whether findings suggest a beneficial effect, a neutral effect, or harm.

## 4 Discussion

This predefined statistical analysis plan provides a comprehensive framework for analyzing the DANOHCA trial, which evaluates four interventions in comatose patients resuscitated from OHCA. The plan was developed and finalized before any outcome data were accessed, minimizing the risk of analytic bias and enhancing transparency.

### 4.1 Strengths

Several features strengthen this analysis plan. First, the use of intervention‐specific primary outcomes acknowledges that different interventions may have different mechanisms and time courses of effect. Mortality outcomes are appropriate for interventions targeting systemic inflammation and cerebral perfusion, whereas days alive outside the hospital capture both survival and functional recovery for interventions targeting sedation and delirium. Second, the factorial design efficiently evaluates four interventions in a single trial, yielding robust estimates of individual intervention effects with the same sample size as a single two‐arm trial. Third, a comprehensive assessment of statistical assumptions ensures a valid inference. Fourth, prespecified subgroup and interaction analyses allow exploration of differential effects across patient populations and potential synergies or antagonisms between interventions. Fifth, independent statistical analyses by two statisticians enhance reproducibility and minimize errors.

### 4.2 Limitations

Several limitations warrant consideration. The analysis plan does not adjust for multiple comparisons across the four interventions, increasing the risk of Type I error. This decision reflects the pragmatic consideration that all four interventions address distinct mechanistic pathways and are justified independently. However, findings will be interpreted cautiously, and interactions will be evaluated if multiple interventions show non‐neutral results. Second, the factorial design assumes no interactions between interventions. While preliminary simulations suggest adequate power even with minor interactions, substantial interactions could reduce power and complicate interpretation. Third, the backrest elevation and early wakeup interventions are unblinded, potentially introducing performance and detection biases. However, primary outcomes (mortality and days alive outside the hospital) are objective and ascertained by blinded assessors. Fourth, the per‐protocol analysis may be affected by selection bias if nonadherence is related to prognosis. Results will be interpreted as exploratory and hypothesis‐generating. Fifth, anticipated intervention effects informing sample size calculations are based on clinical judgment and prior trials, introducing uncertainty. Statistical power may be lower if true effects are smaller.

### 4.3 Clinical Implications

The DANOHCA trial addresses critical gaps in post‐cardiac arrest care. High‐dose corticosteroids may attenuate systemic inflammation and improve hemodynamics. Elevated backrest positioning may reduce intracranial pressure and enhance cerebral perfusion. An early wakeup call may minimize sedation‐related complications while enabling earlier neurologic assessment and rehabilitation. Prophylactic Olanzapine may prevent delirium, which is associated with prolonged hospitalization and worse outcomes. By evaluating these interventions simultaneously in a factorial design, the trial efficiently generates evidence to guide clinical practice.

The use of intervention‐specific primary outcomes reflects the mechanistic rationale for each intervention. Mortality is the most clinically relevant outcome for Dexamethasone and backrest elevation, which target pathways directly affecting survival. Days alive outside the hospital integrates survival and functional recovery, capturing the potential benefits of olanzapine and early wakeup in reducing delirium and facilitating earlier discharge. This approach maximizes clinical relevance while ensuring adequate statistical power.

### 4.4 Methodologic Considerations

The factorial design requires careful attention to potential interactions. If interventions act through independent mechanisms without synergy or antagonism, factorial analysis is efficient and valid. However, if substantial interactions exist, main effects may be misleading. This plan includes rigorous assessment of interactions, with criteria for both statistical significance (*p* < 0.01) and clinical importance (absolute effect ≥ 5.6%) to avoid false‐positive findings. If important interactions are identified, results will be presented with appropriate stratification.

The modified intention‐to‐treat approach balances statistical rigor with clinical pragmatism. Including only participants who meet the inclusion criteria avoids diluting treatment effects from ineligible patients while maintaining randomization integrity. Per‐protocol analyses will provide complementary information about intervention effects under ideal adherence but must be interpreted cautiously given potential selection bias.

Handling of missing data follows current best practices. Registry linkage for mortality ensures complete ascertainment of the primary outcome for Dexamethasone and backrest interventions. Days alive outside the hospital can be ascertained from hospital records and national registries with minimal missingness. Nevertheless, the plan includes multiple imputation and sensitivity analyses to assess the robustness of the findings under different missing‐data assumptions.

## 5 Conclusion

This predefined statistical analysis plan provides a rigorous, transparent framework for analyzing the DANOHCA trial. By specifying outcomes, analysis methods, assumption checks, and sensitivity analyses before data access, this plan minimizes analytic bias and enhances the credibility of findings. The trial will provide high‐quality evidence regarding four interventions targeting distinct mechanistic pathways in post‐cardiac arrest syndrome, with the potential to improve outcomes for comatose survivors of out‐of‐hospital cardiac arrest.

## Author Contributions

Jesper Kjaergaard, Christian Hassager, Jacob E. Moeller, Henrik Schmidt, Jo B. Andreasen, Bodil S. Rasmussen, Anders M. Grejs, Steffen Christensen, Lars P.K. Andersen, and Simon Mølstrøm designed the study protocol of the DANOHCA trial. Together with Sören Möller, these authors wrote the statistical analysis plan. B. Liebetrau and Simon Mølstrøm wrote the first draft of the manuscript. All authors contributed to manuscript editing and approved the final version of the manuscript.

## Funding

The DANOHCA trial has applied for funding by external foundations for medical research and has received an unrestricted research grant of 14.8 million DKK from the Novo Nordisk Foundation, Denmark (application number: 079649), and 3.2 million DKK from the Danish Heart Foundation (grant number: 2023‐12401); neither the Novo Nordisk Foundation nor the Danish Heart Foundation have a financial interest in the study or participated in the design or execution of the trial.

## Conflicts of Interest

Christian Hassager reports research grants from the Lundbeck Foundation, the Novo Nordisk Foundation, and the Danish Heart Foundation; and speakers' honorarium from Abiomed, BD, and Novo Nordisk outside the submitted work. Jacob E. Moeller reports Institutional grants from the Novo Nordisk Foundation and Abiomed; speaker's fee from Abbott and Abiomed; and advisory board membership with Boston Scientific and Magenta. All outside the submitted work. The other authors declare no conflicts of interest.

## Data Availability

Data sharing not applicable to this article as no datasets were generated or analyzed during the current study.

## References

[1] K. G. Hirsch , B. S. Abella , E. Amorim , et al., “Critical Care Management of Patients After Cardiac Arrest: A Scientific Statement From the American Heart Association and Neurocritical Care Society,” Neurocritical Care 40, no. 1 (2024): 1–37. 38040992 10.1007/s12028-023-01871-6 PMC10861627

[2] J. P. Nolan , C. Sandroni , A. Cariou , et al., “European Resuscitation Council and European Society of Intensive Care Medicine Guidelines 2025: Post‐Resuscitation Care,” Intensive Care Medicine 51, no. 12 (2025): 2213–2288. 41123621 10.1007/s00134-025-08117-3

[3] R. W. Neumar , J. P. Nolan , C. Adrie , et al., “Post‐Cardiac Arrest Syndrome: Epidemiology, Pathophysiology, Treatment, and Prognostication. A Consensus Statement From the International Liaison Committee on Resuscitation (American Heart Association, Australian and New Zealand Council on Resuscitation, European Resuscitation Council, Heart and Stroke Foundation of Canada, InterAmerican Heart Foundation, Resuscitation Council of Asia, and the Resuscitation Council of Southern Africa); the American Heart Association Emergency Cardiovascular Care Committee; the Council on Cardiovascular Surgery and Anesthesia; the Council on Cardiopulmonary, Perioperative, and Critical Care; the Council on Clinical Cardiology; and the Stroke Council,” Circulation 118, no. 23 (2008): 2452–2483. 18948368 10.1161/CIRCULATIONAHA.108.190652

[4] J. W. Devlin , Y. Skrobik , C. Gelinas , et al., “Clinical Practice Guidelines for the Prevention and Management of Pain, Agitation/Sedation, Delirium, Immobility, and Sleep Disruption in Adult Patients in the ICU,” Critical Care Medicine 46, no. 9 (2018): e825–e873. 30113379 10.1097/CCM.0000000000003299

[5] A. Ceric , T. L. May , A. Lybeck , et al., “Cardiac Arrest Treatment Center Differences in Sedation and Analgesia Dosing During Targeted Temperature Management,” Neurocritical Care 38, no. 1 (2023): 16–25. 35896768 10.1007/s12028-022-01564-6 PMC9935704

[6] J. Hasslacher , F. Steinkohl , H. Ulmer , et al., “Increased Risk of Ventilator‐Associated Pneumonia in Patients After Cardiac Arrest Treated With Mild Therapeutic Hypothermia,” Acta Anaesthesiologica Scandinavica 66, no. 6 (2022): 704–712. 35338658 10.1111/aas.14063 PMC9321159

